# Improving the Antitumor Activity and Bioavailability of Sonidegib for the Treatment of Skin Cancer

**DOI:** 10.3390/pharmaceutics13101560

**Published:** 2021-09-26

**Authors:** Amr Gamal, Haitham Saeed, Fatma I. Abo El-Ela, Heba F. Salem

**Affiliations:** 1 Department of Pharmaceutics and Industrial Pharmacy, Faculty of Pharmacy, Beni-Suef University, Beni-Suef 625617, Egypt; Amr_g@pharm.bsu.edu.eg; 2 Clinical Pharmacy Department, Faculty of Pharmacy, Beni-Suef University, Beni-Suef 625617, Egypt; Haitham.sedawy@pharm.bsu.edu.eg; 3 Department of Pharmacology, Faculty of Veterinary Medicine, Beni-Suef University, Beni-Suef 625617, Egypt; Fatma.aboel3la@vet.bsu.edu

**Keywords:** skin cancer, Sonidegib, ethosomes, targeting, bioavailability

## Abstract

Throughout the United States and the world, skin cancer is the most frequent form of cancer. Sonidegib (SNG) is a hedgehog inhibitor that has been used for skin cancer treatment. However, SNG has low bioavailability and is associated with resistance. The focus of this work is to enhance bioavailability, anti-tumor efficacy and targeting of SNG via developing ethosome gel as a potential treatment for skin cancer. SNG-loaded ethosomes formulation was prepared and characterized in vitro by %entrapment efficiency (%EE), vesicle size, morphology, %release and steady-state flux. The results showed that the prepared formulation was spherical nanovesicles with a %EE of 85.4 ± 0.57%, a particle size of 199.53 ± 4.51 nm and a steady-state flux of 5.58 ± 0.08 µg/cm^2^/h. In addition, SNG-loaded ethosomes formulation was incorporated into carbopol gel to study the anti-tumor efficacy, localization and bioavailability in vivo. Compared with oral SNG, the formulation showed 3.18 times higher relative bioavailability and consequently significant anti-tumor activity. In addition, this formulation showed a higher rate of SNG penetration in the skin’s deep layers and passive targeting in tumor cells. Briefly, SNG-loaded ethosome gel can produce desirable therapeutic benefits for treatment of skin cancer.

## 1. Introduction

Skin cancer is a malignant epidermal tumor, characterized by the uncontrolled growth of skin cells [1,2]. In the world, the most common kind of cancer is skin cancer, which accounts for at least 40% of all cancer cases [3,4]. Sonidegib (SNG) is a hedgehog inhibitor that has been used for skin cancer treatment [5]. SNG inhibits Smoothened (SMO), which plays a critical role in stem cell maintenance and tissue repair [5]. Despite the fact that SNG has been shown to be effective in the treatment of skin cancer, it has low bioavailability and is associated with adverse effects and resistance [6,7]. The use of nanoparticles holds great promise, both clinically and in pharmaceutical research. Nanoparticles are drug delivery systems which deliver therapeutic agents in a targeted and controlled manner [8,9]. Drug-loaded nanoparticles improve bioavailability, efficacy and selectivity in targeting neoplastic cells [8,9].

Liposomes are the most widely used nanoparticles to treat skin cancer. Liposomes are phospholipid vesicles with an interior aqueous phase [8]. Liposomes are ideal carriers for drug delivery because they have excellent diffusion properties and they can deliver therapeutic agents in a targeted and controlled manner. However, liposomes can provide a sustained and activated release of their payload, but they undergo leakage of encapsulated drug, low stability and low dermal penetration properties [10,11]. To improve the vesicular properties and skin permeability of liposomes, ethosomes vesicles have been introduced. Ethosomes are made up of phospholipids, ethanol (up to 40% *w/w*) and water [12]. Ethosomes have been reported to be superior to liposomes because they have greater entrapment efficiency and better skin stability and permeability characteristics [12,13]. The presence of ethanol improves drug quantity and transdermal flux and improves dermal drug delivery because it is a potent provider of negative charge [2,12].

The transdermal delivery system refers to the method of applying the preparation to the skin to treat diseases such as skin cancer [14]. Compared with the oral route, transdermal application of the drug prevents first-pass metabolism of the liver and fluctuations in plasma levels [15]. In addition, the transdermal drug delivery system can improve drug bioavailability, drug targeting and minimize systemic drug toxicity [16]. There are a variety of topical chemotherapeutics on the market (5-fluorouracil, imiquimod and carmustine) that can be used to treat skin cancer [17]. The aim of this study was to enhance bioavailability, anti-tumor efficacy and targeting of SNG via developing a stable ethosomes formulation of SNG as a potential treatment for skin cancer, and to study the influence of ethanol additives on the physical and chemical properties of liposomes to improve vesicle characteristics and skin penetration of liposomes. The in vitro and in vivo characterizations of the ethosomes formulation were evaluated to investigate its physico-chemical characteristics and anti-tumor efficacy.

## 2. Materials and Methods

### 2.1. Materials

Ethanol, dialysis bags, phospholipon90 G and cholesterol were purchased from Agitech Company, New Cairo, Egypt. Propylene glycol and carbopol 974; other materials were bought from Cornell Lab Company (Cairo, Egypt).

### 2.2. Preparation of Sonidegib Loaded Ethosomes

As described by Aute et al., Sonidegib loaded ethosomes (SLE) formulation was prepared by the hot method [18]. Phospholipon 90 G (3% *w/w*) and cholesterol (0.15% *w/w*) were dispersed in a phosphate buffer (pH 5.5) under vigorous stirring (40 °C, a solution I). Ethanol (40% *v/v*) and propylene glycol (10% *v/v*) were used to dissolve SNG (10 mg) under vigorous stirring (40 °C, a solution II). Drop by drop, solution II was added to solution I under vigorous stirring. The ethosomes were then sonicated for 30 min using an ultrasonicator (Sonix TV, Vernon Hills, IL, USA). The suspended vesicles were stored in the refrigerator (4 °C) for further characterization.

### 2.3. Preparation of Sonidegib Loaded Liposomes

As described by Tefas et al., Sonidegib loaded liposomes (SLL) formulation was prepared by the thin film hydration method [19]. Chloroform was used to dissolve phospholipon 90 G (3% *w/w*), cholesterol (0.15% *w/w*) and SNG (10 mg). Then the solution was evaporated using a rotary evaporator (RE300, Mamhilad, UK) under reduced pressure at 40 °C. The formed film was allowed to be hydrated in isotonic phosphate buffer (IPB, 10 mL, pH 5.5, 40 °C, 1 h). The liposomal suspension was then sonicated for 30 min using an ultrasonicator (Sonix TV, Vernon Hills, IL, USA). The suspended vesicles were stored in the refrigerator (4 °C) for further characterization.

### 2.4. In Vitro Evaluation of SLE and SLL Formulations

#### 2.4.1. Entrapment Efficiency Determination

The HPLC technique was used to quantify SNG [20]. The isocratic separation of SNG was obtained by using a 25 cm × 4.6 mm analytical MG II column C18 at λmax of 254 nm. A 10:10:80 *v/v* mixture of methanol, water and acetonitrile was employed for the mobile phase with a 1 mL/min flow rate and a 20 µL injection volume. The drug content in the prepared formulation was calculated by measuring the entrapment efficiency (%EE). The SLE and SLL suspensions were centrifuged at 15,000 rpm for 1 h at a temperature of 4 °C to separate the entrapped SNG. Methanol was used to dissolve the entrapped SNG and entrapment efficiency was determined using the above HPLC method as follows [21]:

%EE = Et/Ei × 100(1)

where Et is the amount of entrapped SNG and Ei is the initial SNG amount.

#### 2.4.2. Zeta Potential and Particle Size Determination

The SLE and SLL formulations were mixed with distilled water and the average size and zeta potential were measured in three replicates by dynamic light scattering (Malvern, Kassel, Germany) [22].

#### 2.4.3. Thermal Analysis Studies

The thermal analysis of the SNG, phospholipid, cholesterol, SLL and SLE was examined using Differential Scanning Calorimetry (DSC-60F3, NETZSCH-Geratebau GmbH, Maia, Germany) [23]. In addition, DSC detects possible polymorphic transformations during crystallization. The measurements were obtained at a 5 °C/min heating rate with a 25 mL/min nitrogen gas flow rate. This was accomplished by heating the samples from 25 to 250 °C and then rapidly cooling them down to 25 °C.

#### 2.4.4. STEM Measurements

The appearance of the SLE and SLL vesicles was examined using the Scanning Transmission Electron Microscope (STEM, Carl Zeiss, Jena, Germany) [24]. The formulation was adhered to the surface of the carbon-coated copper grid and the film was observed under STEM at suitable magnifications.

#### 2.4.5. Stability Studies

Changes in particle size and EE of SLE and SLL formulations were investigated to determine the stability studies of SLE and SLL formulations [12]. The prepared formulations were stored at different temperatures (4 °C, 25 °C and 40 °C) for 3 months, and every month, samples were characterized for size and EE in three replicates.

#### 2.4.6. In Vitro Drug Release Studies

The saturated solubility of SNG was measured by the equilibrium solubility study using HPLC in triplicate to determine the dissolution medium volume that best meets the sink conditions of the SNG release study from the ethosomal formulation [25]. The solubility of SNG in phosphate buffer (pH 5.5) was determined by mixing excess SNG with 2 mL of phosphate buffer in a 5 mL vial with a stopper using a vortex mixer for 72 h at 25 ± 1.0 °C.

The release of SNG from SLE, SLL and free SNG suspension was determined using the Hanson dissolution apparatus [26]. A volume of SLE, SLL and free SNG suspension equivalent to 1 mg of SNG was administered in the dialysis bag which was then immersed in 50 mL of phosphate buffer (pH 7.4) containing 0.1% *w/w* of Tween 80. The dissolution apparatus (Hanson, USA) was adjusted at 100 rpm and 37 ± 0.5 °C. Samples of 3 mL were removed from each receptor compartment and were replenished with an equal volume at predetermined time points. The % release was determined using the above HPLC as follows in triplicate.

%Release = Rt/Ri × 100(2)

where Rt is the amount of SNG released at time t and Ri is the initial amount of entrapped SNG.

#### 2.4.7. Drug Release Kinetics

The in-vitro release kinetics of SLE was measured using DDSolver program software [27,28]. A built-in model library of 40 dissolution models is used in the DDSolver software to model dissolution data using nonlinear optimization methods. The coefficient of determination (R^2^), Akaike Information Criterion (AIC) and Model Selection Criterion (MSC) criteria were used to assess a model’s goodness of fit. The model fitting the release mechanism of SNG is the model that gives the highest R^2^, lowest AIC and highest MSC. Similarly, the mechanism of SNG release was also studied using the Korsmeyer-Peppas equation [29]. If *n* = 0.5, it generally means that Fickian diffusion is the mechanism of release; and if 0.5 < *n*< 1, it generally means that non-fickian diffusion is the mechanism of release. In addition, the DDSolver program implements different functions to calculate the similarity factor *f_2_* to predict the difference between the SNG released by SLE and the free SNG. If *f_2_* >50, it generally means that the difference between the SNG released by SLE and the free SNG is insignificant (*p* < 0.05); and if *f_2_* < 50, it generally means that the difference between the SNG released by SLE and the free SNG is significant (*p* < 0.05).

#### 2.4.8. Ex Vivo Drug Permeation and Skin Deposition Studies

A Hanson diffusion cell (5 cm^2^) with Guinea pig skin as a donor compartment was used to measure the in vitro permeation of SNG from SLE, SLL formulations and free SNG suspension [30]. The receptor compartment was filled with 50 mL of phosphate buffer (pH 7.4) containing 0.1% *w/w* of Tween 80 as a receptor medium to comply with the sink condition. The dissolution apparatus (Hanson, Santa Clara, USA) was adjusted at 100 rpm and 37 ± 0.5 °C. A volume of SLE, SLL formulations and free SNG suspension equivalent to 1 mg of SNG was administered in the donor compartment, which was then immersed in the receptor compartment. Samples of 3 mL were removed from each receptor compartment and were replenished with an equal volume at predetermined time points. The permeation samples were analyzed using the above HPLC method. The transdermal flux (Fss) was calculated in triplicate as follows:

Fss = (The permeation rate)/(The active diffusion area)(3)

After termination of the permeation study, the skin was chopped into small pieces mixed with phosphate buffer (pH 7.4) and 0.1% *w/w* Tween 80 to determine the skin deposition of SNG from SLE, SLL formulations and free SNG suspension [31]. The skin pieces were homogenized under homogenization at 8000 rpm using a high-shear homogenizer (DI 25 basic, IKA Germany, Staufen, Germany) for 10 min to ensure the complete release of SNG. The skin homogenate was centrifuged at 10,000 rpm for 5 min and analyzed using the above HPLC method in triplicate.

### 2.5. Preparation and In Vitro Characterization of SLE and SLL Gel Formulations

#### 2.5.1. Preparation of SLE and SLL Gel Formulations

A Carbopol 974 (2% *w/w*) was used to prepare the gel base by dispersing Carbopol 974 under vigorous stirring in water [12]. In order to adjust the pH of the gel base, triethnolamine was used. Incorporating free SNG into the Carbopol gel base under vigorous stirring produced the free SNG gel, while incorporating SLE and SLL formulations into the Carbopol gel base under vigorous stirring produced the SLE gel and SLL gel formulations, respectively [32]. After preparation, the gel formulations were refrigerated at 4 °C.

#### 2.5.2. In Vitro Evaluation of SLE and SLL Gel Formulations

The viscosity coefficient of the prepared gel formulations was measured using a Brookfield viscometer (DV-III, AMETEK Brookfield, Middleborough, MA, USA) [24]. From the log shear rate versus log shear stress, the viscosity coefficient was calculated using the following formula:

Log (shear stress) = N log (shear rate) − log(viscosity coefficient) (4)

The permeation of prepared gel formulations was measured in vitro as described previously.

### 2.6. In Vivo Anti-Tumor Characterization of SLE Gel Formulation

#### 2.6.1. Study Design

In this case, 48 adult male mice (200–300 g) were housed with a balanced diet and water at 22 ± 2 °C and a humidity of 50 ± 5%. The mice were acclimatized for one week before being used for experimentation and allowed for standard conditions such as free water access, diet and clean cages. Each animal’s dorsal skin was clipped 48 h before the experiment began in order to remove a 3 × 3 cm^2^ area. A single dosage of the tumor initiator DMBA (1 mg in 200 μL acetone) was given subcutaneously to each mouse to generate a tumor [33]. Increased epidermal tumors which were larger and progressed more frequently to malignant carcinoma known as papillomas were observed after the injection of DMBA. The animal ethical committee of Beni-Suef University’s Faculty of Pharmacy gave its approval to this procedure.

#### 2.6.2. Animals

Mice were randomly separated into five groups, each with six individuals. The first group was reserved as a positive control group. The second group was treated orally with free SNG suspension. The third group was treated topically with free SNG gel. The fourth group was treated topically with SLL gel formulation. The fifth group was treated topically with SLE gel formulation.

#### 2.6.3. Anti-Tumor Activity and Toxicity Determination

Histopathology and a standard method of measuring the number and diameter of papilloma were used to investigate the antitumor activity and toxicity of a particular treatment [33]. Papillomas larger than 1 mm in diameter were counted and recorded weekly until the study was completed. At the end of the experiment, all mice among different groups were anaesthetized and sacrificed. Buffered formalin was used to fix the tumor samples, which were then cut and stained with hematoxylin-eosin for histopathological examination [34].

#### 2.6.4. In Vivo Permeation and Bioavailability Studies

The effect of ethanol additive on the skin permeability of liposomes was confirmed in vivo by comparing the skin permeation of the SLE gel formulation with that of the SLL gel. In this case, 18 adult male mice (200–300 g) were randomly separated into three groups, each with six individuals. The first group was treated orally with free SNG suspension. The second group was treated topically with SLL gel formulation. The third group was treated topically with SLE gel formulation. Blood samples at different time intervals for 24 h after administration were collected in EDTA tubes and centrifuged at 3.0× *g* for 10 min, followed by separation of plasma till analyzed by the above HPLC method. Plasma samples were mixed with acetonitrile and centrifuged at 3.0× *g* for 10 min. The supernatant was vaporized and dissolved in the mobile phase to be analyzed to determine the total amount of SNG permeated by HPLC in triplicate [35]. The WinNonlin^®^ software (version 1.5, NJ, USA) was used to perform the non-compartmental analysis) [26]. The linear trapezoidal method was used to measure the area under concentration time curve (AUC). The plasma concentration vs. time profile was obtained to determine the maximum concentration (Cmax) and mean residence time (MRT). The statistical analysis was measured using the student’s *t*-test.

For the second and third groups, skin sections were cut and stored at −80 °C to determine how much SNG remained in the skin. The tape stripping technique was used for removing the stratum corneum and the skin was homogenized under homogenization at 8000 rpm using a high-shear homogenizer (DI 25 basic, Germany) for 10 min [36]. The tissue homogenate (1 mL) was mixed with acetonitrile and centrifuged at 3.0× *g* for 10 min. The supernatant was vaporized and dissolved in the mobile phase to be analyzed to determine the drug concentration by HPLC in triplicate [33]. The statistical analysis was measured using the student’s *t*-test.

### 2.7. Statistical Analysis

The ANOVA test (*p* < 0.05) was used for all statistical analysis of the data using Design-Expert^®^ software and IBM-SPSS Statistics (version 22, USA). The data were presented as the mean ± standard deviation (SD).

## 3. Results

### 3.1. Preparation and In Vitro Characterization of SLE and SLL Formulations

#### 3.1.1. Entrapment Efficiency Determination

Successfully, SLE formulation containing concentrations of phospholipon 90 G (3% *w/w*), cholesterol (0.15% *w/w*) and ethanol (40% *v/v*) [2,12,37] and SLL formulation containing concentrations of phospholipon 90 G (3% *w/w*) and cholesterol (0.15% *w/w*) [38,39,40] were prepared. The HPLC method was used to quantify SNG. Linearity was obtained with a coefficient of determination (R^2^) of 0.999 and a retention time of 4.95 min. The EE of SLE and SLL formulations was determined and found to be 85.4 ± 0.57% and 75.27 ± 0.91%, respectively.

#### 3.1.2. Zeta Potential and Particle Size Determination

**Figure 1:**
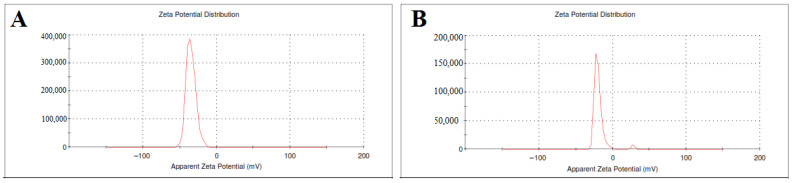
The zeta potential of SLE (**A**) and SLL formulations (**B**).

The zeta potential of SLE and SLL formulations was measured as shown in Figure 1 and found to be −44.5 ± 0.74 mV and −20.63 ± 0.38 mV, respectively.

**Figure 2:**
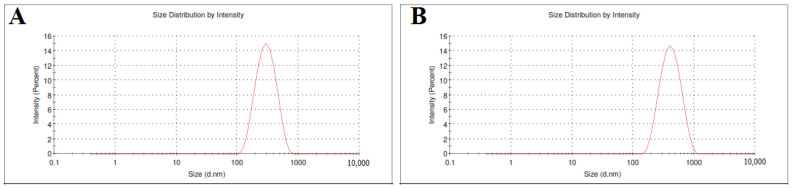
The particle size of SLE (**A**) and SLL formulations (**B**).

The particle size of SLE and SLL formulations was measured as shown in Figure 2 and found to be 199.53 ± 4.51 nm and 328.87 ± 2.48 nm, respectively, with a low polydispersity index of 0.206 ± 0.02 and 0.231 ± 0.03, respectively.

#### 3.1.3. Thermal Analysis Studies

**Figure 3:**
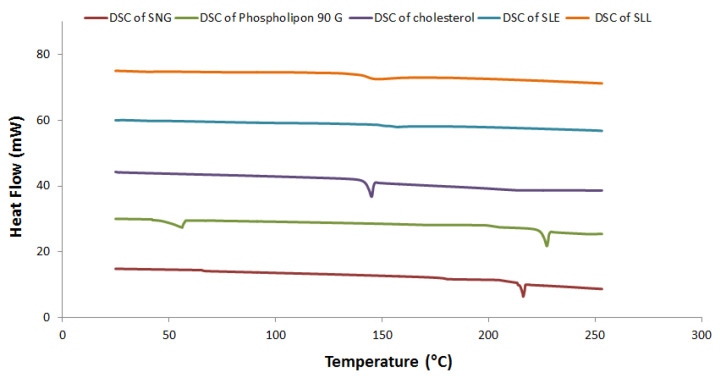
DSC thermogram of SLE and SLL formulations components.

Figure 3 shows the crystallization characteristics and thermal behavior of SNG, phospholipon 90 G, cholesterol, SLE and SLL formulations using DSC. The DSC studies showed that the endothermic peak of SNG was located at 214.51 °C with an enthalpy fusion (ΔH) of −63.10 mJ, the endothermic peaks of phospholipon 90 G were located at 51 °C and 224 °C with an enthalpy fusion (ΔH) of −85.64 mJ and the endothermic peak of cholesterol was located at 144.46 °C with an enthalpy fusion (ΔH) of −105.91 mJ. When SNG, cholesterol and phospholipon 90 G were mixed together, their endothermic peaks were diminished.

#### 3.1.4. STEM Measurements

**Figure 4:**
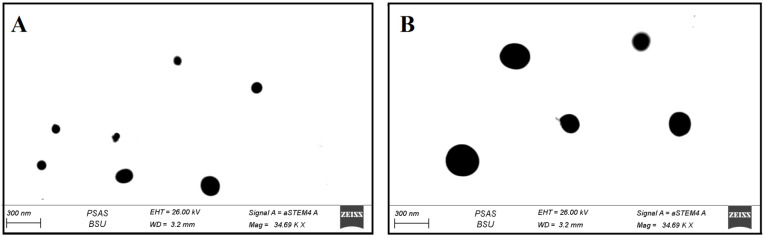
Surface morphology of SLE (**A** and **B**) and of SLL (**B**) by STEM.

According to the STEM, Figure 4 displays the SLE and SLL formulations surface morphology. Both formulations are spherical nano-vesicles that appear as black dots.

#### 3.1.5. Stability Studies

**Figure 5:**
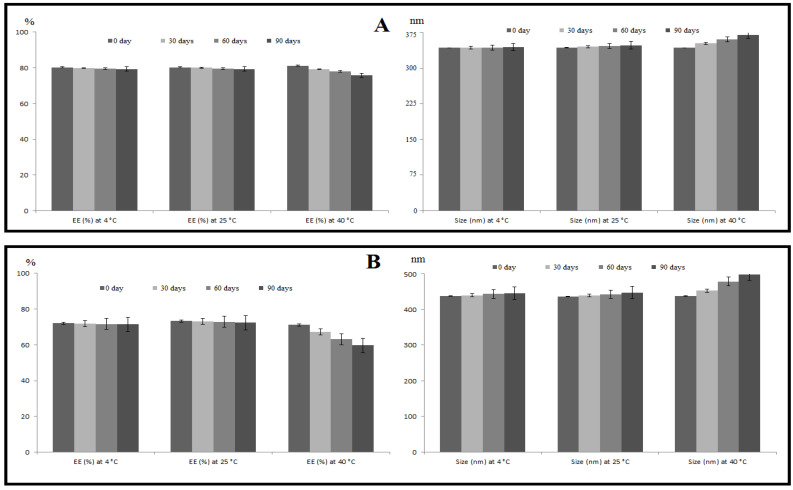
Effect of storage on the %EE and particle size of SLE (**A**) and SLL (**B**) formulations at 4 °C, 25 °C and 40 °C. Each value was the mean ± standard deviation of measurements from three samples.

The stability of the SLE and SLL formulations was evaluated as shown in Figure 5. On the ANOVA test (*p* > 0.05), the SLE formulation showed an insignificant decrease in EE and an insignificant increase in size at 4 °C, 25 °C and 40 °C. The SLL formulation showed an insignificant (*p* > 0.05) decrease in EE and an insignificant (*p* > 0.05) increase in size at 4 °C and 25 °C, but at 40 °C it showed a significant (*p* > 0.05) decrease in EE and a significant (*p* > 0.05) increase in size.

#### 3.1.6. In Vitro Drug Release and Ex Vivo Permeation Studies

**Figure 6:**
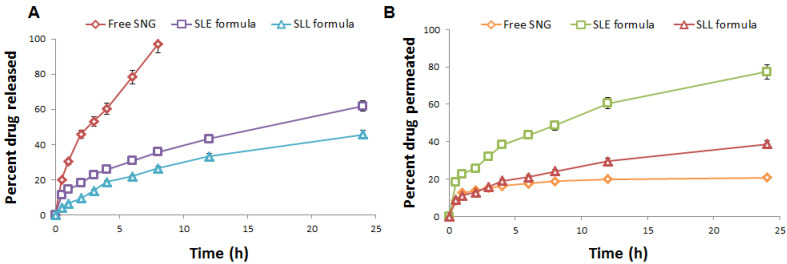
(**A**) In vitro release profile and (**B**) In vitro permeation profile of SNG from the prepared formulations (*n* = 3 ± SD).

The equilibrium solubility test was obtained and the saturated solubility of SNG was found to be 0.02 mg/mL. The phosphate buffer (50 mL, pH 5.5) containing 0.1% *w/w* of Tween 80 was selected as the dissolution medium. As shown in Figure 6, the release of SNG from SNG suspension was significantly (*p*-value < 0.05) higher than that of SLE and SLL formulations, where the release of SNG from SNG suspension, SLE and SLL formulations was found to be 97.07 ± 0.52%, 61.87 ± 0.57% and 45.83 ± 0.62%, respectively. The permeation of free SNG was significantly (*p*-value < 0.05) lower than that of SLE and SLL formulations, where the permeation of SNG from SNG suspension, SLE and SLL formulations was found to be 41.57 ± 1.40 µg/cm^2^, 154.74 ± 1.72 µg/cm^2^ and 77.35 ± 0.89 µg/cm^2^, respectively. The steady-state flux of SNG from SNG suspension, SLE and SLL formulations was found to be 1.42 ± 0.05 µg/cm^2^/h, 5.58 ± 0.08 µg/cm^2^/h and 2.71 ± 0.05 µg/cm^2^/h, respectively. The skin deposition of SNG from SNG suspension, SLE and SLL formulations was found to be 155.46 ± 1.53 µg/cm^2^, 48.42 ± 2.55 µg/cm^2^ and 119.44 ± 2.06 µg/cm^2^, respectively.

#### 3.1.7. Drug Release Kinetics

DDSolver software was used to model dissolution data and simplify evaluating the similarity between dissolution profiles [27,28]. As shown in Table 1, the Korsmeyer-Peppas model was found to be appropriate to describe the SNG release from SLE formulation because it gave the maximum values of R^2^ and MSC of 0.9981 and 5.7188, respectively, and the minimum value of AIC of 17.7495. To determine the mechanism of SNG release from SLE, the value of “n” was calculated from the Korsmeyer-Peppas equation and was found to equal 0.441 ± 0.007, which indicates that the drug is released in a Fickian diffusion way. The Similarity factor (*f_2_*) was calculated using DDSolver to compare the SNG release profile from SLE with that of free SNG. The *f_2_* was found to equal 32.12 ± 0.87 which revealed the differences in dissolution profiles with a significant difference of *p* < 0.05 and the ethosomes have a major impact on SNG release behavior.

### 3.2. Preparation and In Vitro Characterization of SLE and SLL Gel Formulations

**Figure 7:**
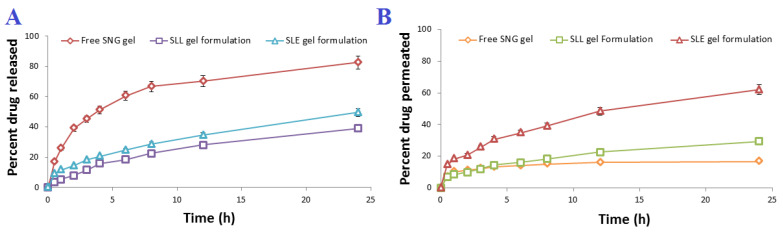
(**A**) In vitro release profile and (**B**) In vitro permeation profile of SNG from the prepared gel formulations (*n* = 3 ± SD).

The SLE and SLL formulations were successfully integrated into a Carbopol gel. The viscosity coefficient of SLE, SLL gel formulations and free SNG gel was measured and found to be 178.98 ± 0.62, 182.34 ± 0.57 cP and 186.89 ± 0.57 cP, respectively. As shown in Figure 7, incorporation of SLE and SLL into Carbopol gel significantly (*p* < 0.05) retarded the release of SNG up to 49.83 ± 0.62% and 39.27 ± 0.83%, respectively, within 24 h and the permeation of SNG, where the amount of SNG permeated from SLE and SLL gel formulations was 124.43 ± 2.12 µg/cm^2^ and 59.15 ± 1.92 µg/cm^2^, respectively. The mucosal flux of SNG from SLE and SLL gel formulations was found to be 4.58 ± 0.03 µg/cm^2^/h and 2.12 ± 0.05 µg/cm^2^/h, respectively with skin deposition of 85.23 ± 1.42 µg/cm^2^ and 135.23 ± 1.42 µg/cm^2^, respectively.

### 3.3. In Vivo Anti-Tumor Characterization of SLE Gel Formulation

#### 3.3.1. Anti-Tumor Activity and Toxicity Determination

**Figure 8:**
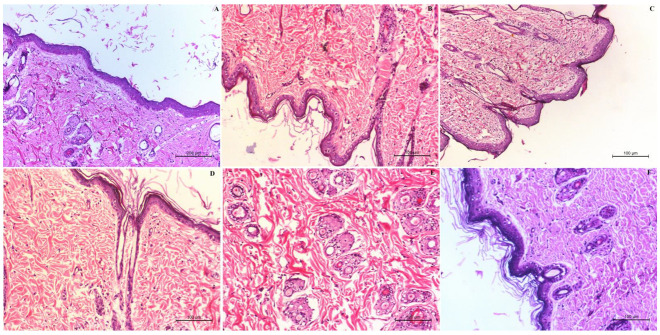
Histological examination of the −ve control group (**A**), +ve control group (**B**), group treated with free SNG gel (**C**), group treated with oral SNG suspension (**D**), group treated with SLL gel (**E**) and group treated with SLE gel (**F**).

A standard method of measuring the number and diameter of papilloma was used to investigate the antitumor activity of the SLE gel formulation. A significant (*p* < 0.05) decrease in the number and diameter of papillomas was observed with the SLE gel when compared to oral SNG suspension and SLL gel. Histopathological examination of the +ve control group (Figure 8B) showed the presence of neoplastic proliferating epithelial cells. Some epithelial cells in this group were vacuolated. Vascular proliferation appeared and caused dermal granulation tissue. All the signs of damage and toxicity in the skin layers appeared with subcutaneous edema in the dermis, besides the hyperkeratosis and inflammatory cell infiltrations. There was no improvement in the signs of skin toxicity either in the dermis or the epidermal layer observed in the group treated with free SNG gel (Figure 8C). The size and number of papilloma still increased even after treatment with oral SNG (Figure 8D), but at slightly lower rates than those of the +ve control group. The surface epithelium of the epidermis showed hyperkeratosis and acanthosis with diffused inflammatory reaction and edema in the dermal subcutaneous. Hyalnosis of some blood vessels (Angiopathic) was indicative of the presence of a chronic proliferative reaction. The group treated with SLL gel (Figure 8E) showed a moderate improvement in the skin toxicity in the size and number of papilloma. Even though Figure 8E showed improvement in the number and infiltration rates of the inflammatory cells, hyperplasia in the skin still appears. The group treated with SLE gel (Figure 8F) showed a marked decrease in hyperplasia and hyperkeratosis when compared to the group treated with SLL gel. The group treated with SLE gel showed a marked absence of inflammatory reactions within the dermis and marked improvement in the skin toxicity with normal architecture, structure and appearance in both the epidermal and dermal layers compared to the –ve control group (mice received neither DMBA nor SNG, Figure 8A).

#### 3.3.2. In Vivo Permeation and Bioavailability Studies

**Figure 9:**
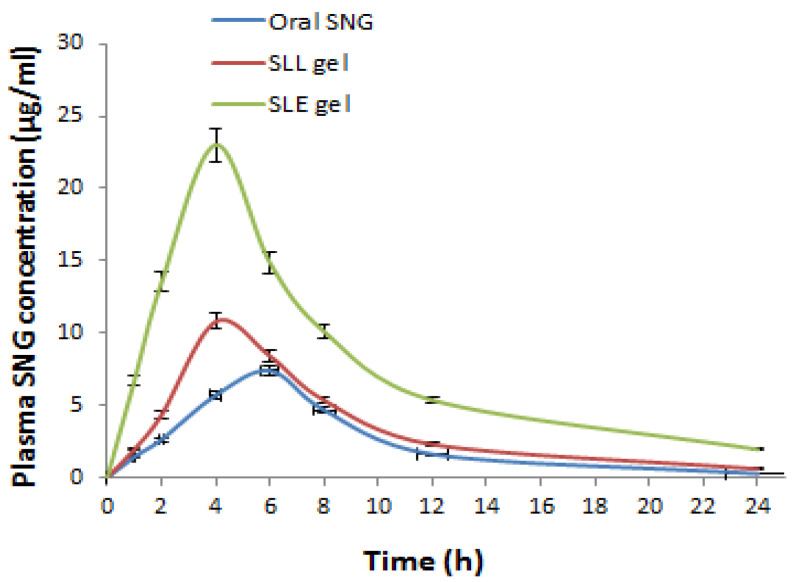
Plasma SNG concentration (μg/mL) after oral administration of free SNG and transdermal administration of SLE and SLL gel formulations.

The plasma concentration-time curve (Figure 9) was drawn to calculate the pharmacokinetic parameters. The AUC for SLE gel (210.80 ± 7.83 μg·h/mL) was significantly (*p* < 0.001) greater than the AUC of oral SNG (66.14 ± 4.53 μg·h/mL) by 3.18 folds. The AUC for SLE gel was significantly (*p* < 0.001) greater than the AUC of SLL gel (90.04 ± 5.52 μg·h/mL) by 2.34 folds. The elimination rate constant (K) for SLE gel (0.0547± 0.05 h^−1^) was significantly (*p* < 0.001) lower than that of oral SNG (0.132 ± 0.07 h^−1^). The half-life (t_0.5_) for SLE gel (12 ± 1.54 h) was significantly (*p* < 0.001) higher than that of oral SNG (5.25 ± 0.82 h). The MRT for SLE gel (23 ± 1.90 h) was significantly (*p* < 0.001) higher than that of oral SNG (13.25 ± 1.31 h).

**Figure 10:**
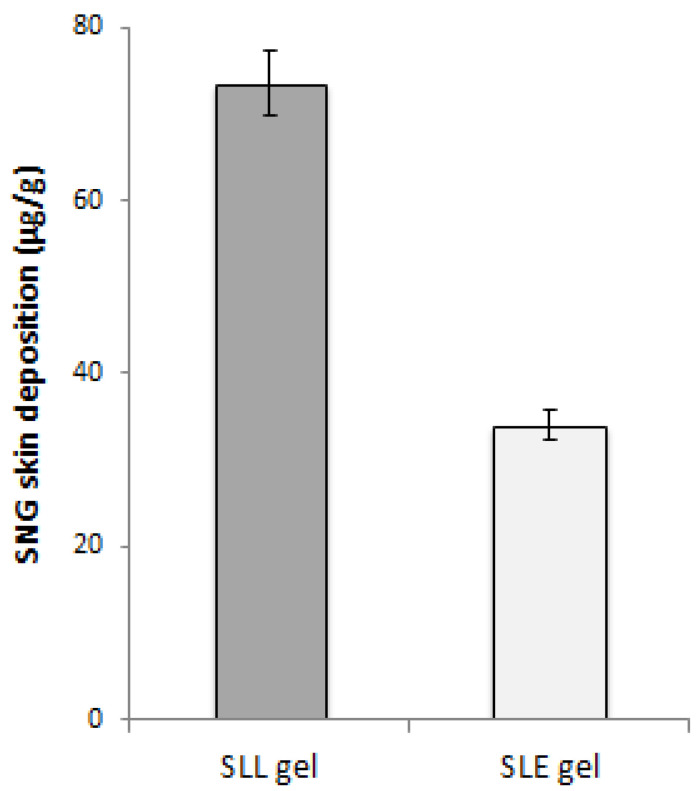
SNG concentration (μg/mL) in the skin after application of SLE and SLL gel formulations.

The impact of ethanol additive on the liposome’s permeation was also confirmed in vivo by comparing the skin deposition of SLE gel formulation with that of SLL gel. The total amount of SNG retained in the skin of mice groups treated with SLE gel and SLL gel was determined. The SLL gel obtained a significant (*p* < 0.05) higher skin deposition concentration than that of SLE gel as shown in Figure 10.

## 4. Discussion

Liposomes are a promising method to improve drug delivery, especially for anticancer drugs [39]. They can increase bio-distribution and prolong circulation time. However, they have low dermal penetration properties, as proven by confocal microscopy [10,11]. To improve the dermal penetration properties of liposomes, ethosomes vesicles have been introduced. Ethosomes are made up of phospholipids, cholesterol and ethanol (up to 40% *w/w*) [12]. Phospholipids are lipid bilayers and are responsible for the formation of multiple layers of vesicles [41,42]. Cholesterol is a rigid molecule that can provide rigidity to the bilayer and improve its physical stability [2,12]. Ethanol is a penetration enhancer and negatively charged provider, giving an increased degree of flexibility for the vesicles thus, improving the drug permeation and targeting in tumor cells [2,12,30,37]. According to literature reviews, preliminary trials were carried out to determine the optimum formulation of SLE and SLL. According to preliminary trials, the presence of phospholipid at a concentration greater than 3% *w*/*w* produced larger-size vesicles with no effect on entrapment efficiency. Cholesterol concentration greater than 0.3% *w/w* produces vesicles with low entrapment efficiency. Ethanol concentration greater than 40% produces vesicles with low %EE and large size. Furthermore, propylene glycol was used at a concentration of 10% *v/v* to enhance skin penetration and significantly decrease the particle size of ethosomes [18]. According to literature reviews and preliminary trials, a formulation containing concentrations of phospholipon 90 G (3% *w/w*), cholesterol (0.15% *w/w*) and ethanol (40% *v/v*) was selected as the optimum formulation of ethosomes [2,12,37] and a formulation containing concentrations of phospholipon 90 G (3% *w/w*) and cholesterol (0.15% *w/w*) was selected as the optimum formulation of liposomes [38,39,40]. Successfully, SLE and SLL formulations were prepared.

The %EE was investigated to calculate the drug content of the prepared formulations. The high %EE of SNG in SLE formulation compared to SLL formulation could be explained by the presence of ethanol which improves drug solubility and distribution within the vesicle, therefore, higher entrapment efficiency [2,12]. The results collected from Dynamic Light Scattering showed a low polydispersity index, indicating a homogeneous distribution of vesicles. The SLE size was smaller than that of the SLL due to the presence of ethanol. Ethanol lowers the thickness of the membrane by creating a phase with interpenetrating hydrocarbon chains and increases the membrane’s dielectric constant and ion permeability. Additionally, ethanol increases the negative charge of vesicles, resulting in increased electrostatic repulsion and decreased aggregation of the vesicles [12,43,44]. Using DSC, you can determine the crystalline characteristics and how much enthalpy a material has changed due to changes in its physical and chemical properties over time [43]. Using this procedure, you may identify and characterise materials. DSC thermograms of SNG, cholesterol and phospholipon 90 G showed melting endothermic peaks which diminished when they were mixed together as a result of SNG’s amorphous integration into the bilayer structure. The surface morphology of SLE and SLL was determined using STEM. When compared to liposomes, the size of ethosomes formulation was smaller, as explained before. Additionally, the stability of SLE and SLL formulations were evaluated. When compared to liposomes, the ethosomes formulation was more stable due to the presence of ethanol [2,12]. The significant increase in SLL size could be explained by the swelling and aggregation of liposomes.

The equilibrium solubility test was obtained to calculate the saturated solubility of SNG and determine the volume of dissolution medium best suited to the sink condition. The phosphate buffer (50 mL, pH 5.5) +0.1% *w/w* of Tween 80 was selected as the dissolution medium because it has a higher solubility in relation to saturation solubility of SNG. The release of SNG from SNG suspension was significantly higher than that of SLE and SLL formulations, while the permeation of free SNG was significantly lower than that of SLE and SLL formulations due to the presence of phospholipid and cholesterol [2,12,42]. The presence of phospholipid and cholesterol in SLE and SLL formulations prevents leakage and reduces permeability and fusion of vesicles [42]. Compared to the SLL formulation, the presence of ethanol in the SLE formulation improves the drug release and permeation. Ethanol is a penetration enhancer and negatively charged provider, giving an increased degree of flexibility for the vesicles, thus improving the drug permeation, absorption and targeting in the tumor cells [12]. DDSolver software helps to use non-linear optimization methods to model dissolution data and simplify evaluating the similarity between dissolution profiles [27,28]. The Korsmeyer-Peppas model was found to be appropriate to describe the SNG release from SLE formulation with a Fickian diffusion drug release mechanism. As evident from in vitro dissolution profiles, these findings predicted drug release ability and vesicle efficacy in delaying SNG release.

Carbopol is an anionic polymer with good buffering capacity which helps maintain the desired pH and does not cause skin irritation [45,46,47]. When combined with ethosomes, Carbopol polymer provides the necessary viscosity and bio-adhesive characteristics [2,12]. The SLE and SLL formulations were successfully integrated into a Carbopol gel. The presence of ethanol could explain the small drop in viscosity coefficient of the SLE gel compared to that of the SLL gel formulation and free SNG gel. Incorporation of SLE and SLL into Carbopol gel significantly retarded the release and permeation of SNG due to Carbopol gel’s cross-linking [46]. A standard method of measuring the number and diameter of papilloma and histopathological examination were used to investigate the antitumor activity of the SLE gel formulation. The group treated with SLE gel showed a significant decrease in the number and diameter of papilloma when compared with other groups. A histopathological examination was obtained to confirm the anti-tumor activity of the SLE gel. Compared to oral SNG and SLL gel at the same dose, SLE gel exhibited enhanced anti-angiogenic and anti-tumor activity. When SNG was incorporated into the ethosomes gel carriers, the papilloma could be elucidated due to the targeting ability and prolonged action of ethosomes [35]. Additionally, histopathological examination was used to investigate the skin toxicity of SLE gel. The results suggested that the dose regimen used did not present significant toxicity in healthy animals.

The pharmacokinetic parameters were calculated and the relative bioavailability of SLE gel was greater than that of oral SNG due to avoiding first-pass hepatic metabolism. Compared to oral SNG, the values of Tmax and Cmax of the SLE gel formulation showed drug release in a controlled manner, reducing the unwanted concentration of SNG. Additionally, a significant (*p* < 0.001) increase in the values of MRT and t_0.5_ for SLE gel compared to that for oral free SNG suspension formulations reflects the longer blood circulation time. These findings confirmed the reduction in the toxicity and unwanted effects of SNG. Compared to the SLL gel formulation, a significant (*p* < 0.001) increase in the values of AUC, Cmax, MRT and t_0.5_ of the SLE gel formulation showed the impact of ethanol additive on the liposome’s permeation, which was confirmed in vivo using the skin deposition studies. Compared to the SLL gel formulation, the SLE gel formulation showed a significantly lower skin deposition concentration due to the presence of ethanol.

## 5. Conclusions

Sonidegib was incorporated into a formulation containing concentrations of phospholipon 90 G (3% *w/w*), cholesterol (0.15% *w/w*) and ethanol (40% *v/v*) to enhance vesicle characteristics and skin penetration of liposomes. Stable spherical nano-vesicles were prepared with %EE of 85.4 ± 0.57% and SNG permeation of 154.74 ± 1.72 µg/cm^2^ with steady-state flux of 5.58 ± 0.08 µg/cm^2^/h. These findings showed that developing SNG-loaded ethosomes formulation plays a significant role in enhancing the permeability of SNG for anticancer delivery. The SLE formulation was then incorporated into Carbopol gel to enhance the bioavailability, anti-tumor efficacy and targeting of SNG as a potential treatment for skin cancer. The SLE gel formulation was evaluated in vivo and showed an increase in SNG dermal permeation and targeting of the tumor cells. Compared with oral SNG, the SLE gel formulation showed greater relative bioavailability and, as a result, significant anti-tumor activity. From our current results, it is clear that SNG loaded ethosomes gel can produce desirable therapeutic benefits for effective treatment of skin cancer with lower side effects.

## Figures and Tables

**Table 1:** DDSolver release data kinetic models to assess the release behavior of SNG from SLE.

Release Data Models	Parameters of Goodness of Fit		
	R^2^	MSC	AIC
Zero-order	0.4626	0.1049	75.2581
First-order	0.7889	1.0393	65.9145
Higuchi	0.9888	3.9877	36.4306
**Korsmeyer-Peppas **	**0.9985**	**5.8935**	**17.3727**
Hixson-Crowell	0.7074	0.7131	69.1770
Hopfenberg	0.7624	0.8388	67.9192
Baker-Lonsdale	0.9981	5.7443	18.8641
Makoid-Banakar	0.9984	5.7804	18.5038
Peppas-Sahlin	0.9984	5.7623	18.6845
Quadratic	0.8658	1.4100	62.2076
Weibull	0.9950	4.6472	29.8352

R^2^: Coefficient of determination; AIC: Akaike Information Criterion; MSC: Model Selection Criterion.

## Data Availability

The data presented in this study are available on request from the corresponding author.
